# Photoluminescence from Two-Phase Nanocomposites Embedded in Polymers

**DOI:** 10.3390/mi15010111

**Published:** 2024-01-09

**Authors:** Mithun Bhowmick, James Christensen, Richard Adjorlolo, Bruno Ullrich

**Affiliations:** 1 Mathematical and Physical Sciences, Miami University Regionals, Middletown, OH 45042, USA; 2 Construction Engineering Research Laboratory, United States Army Corps of Engineers, Champaign, IL 61822, USA; 3 Ullrich Photonics LLC, Manistique, MI 49854, USA

**Keywords:** quantum dots, composites, CdSe, photoluminescence, PVA, nanoparticles, polymers

## Abstract

A set of polymer-embedded, two-colored nanocomposites were prepared where the co-existing emission peaks (~578 nm and ~650 nm) had different ratios at their emission thresholds. The nanocomposite samples were simultaneously excited by a 405 nm laser, and the growth of photoluminescence intensities was studied as a function of excitation intensity. The two peaks showed different growth evolution mechanisms. The factors impacting this difference could be (1) energy transfer between the two sized nanoparticles; (2) relaxation mechanism of smaller nanoparticles; and (3) material properties of the polymer.

## 1. Introduction

Nanotechnology has emerged as a groundbreaking field with diverse applications across various disciplines [1]. Among the nanoscale materials, quantum dots (QDs) hold a special place due to their unique properties [1,2,3,4,5,6,7,8,9,10,11,12]. The convergence of physics, materials science, chemistry, and biotechnology at the nanoscale has paved the way for the creation of novel materials exhibiting properties markedly different from their bulk counterparts [1,2,3,4,5,6,7,8]. In physics and material science, QDs primarily explore the behavior of electrons and photons at the nanoscale, whereas in chemistry, this they are associated with colloids, micelles, polymer composites, and similar structures [1,2,3,4,5,6,7,8,9,10,11,12]. The interdisciplinary nature of nanotechnology allows for the manipulation and design of materials at the atomic and molecular levels, opening new possibilities in fields such as information technology, environmental science, medicine, food safety, agriculture, and more [4,5,6,7,8,9,10,11,12]. Semiconductor QDs, being a pivotal class of materials, hold significant promise for various nanoscale applications owing to their unique structural, optical, and electrical properties [1,2,3,4,5,6,7,8,9,10,11,12]. The distinct characteristics of semiconductors make them particularly exciting for researchers and engineers working on nanoscale technologies. The efforts invested in the synthesis and characterization of semiconductor QDs from groups II–VI, III–V, I–III–VI, and oxide semiconductor materials have been substantial over the past few decades [1,6,7,8]. Among these, wide-band-gap II–VI semiconductor materials have emerged as an important class, showcasing properties that are advantageous for a wide range of novel applications [1,2,3,6].

It is noteworthy that the applications of nanotechnology in fields such as information technology, medicine, and energy continue to evolve as researchers uncover new possibilities and refine existing technologies [1,2,3,4,5,6,7,8,9,10,11,12]. The ability to engineer materials at the nanoscale has not only opened avenues for innovation but has also raised important considerations related to safety, ethics, and environmental impact, which researchers and policymakers need to address as the field progresses. It is probably relevant to summarize the features of QDs systems in this context. 

QDs, or nanoparticles, are nano-sized three-dimensional structures where electrons and holes are confined within dimensions defined by their respective De Broglie wavelengths. The combination of the high surface-to-volume ratio and quantum confinement effect plays a crucial role in shaping their electronic, magnetic, optical, and catalytic properties, compared to the bulk materials or isolated atoms [1]. These altered properties open new possibilities for engineering materials with tailored characteristics, enabling innovative applications across a spectrum of scientific and technological domains. The energy levels of these structures are quantized, leading to behaviors akin to artificial atoms. Over the past four decades, researchers have extensively investigated QD systems due to their appealing optical characteristics. The tunability of narrow-band photoemission based on size, high quantum yield, and the benefits of nanometer dimensions have consistently intrigued scientists. Continuous research attention has expanded the potential applications of QD systems, unveiling new synthesis methods and applications.

QD systems often needed to be used in various flexible applications in a variety of landscapes and geometries [13]. There are situations where direct application of QDs would lead to aggregation, often resulting in degradation of optical properties [14,15,16]. A potential solution to this problem is to use composite nanomaterials. Composite nanomaterials contain more than one component and are used in scenarios where flexibility as well as a combination of component properties are needed. In many cases, QDs are dispersed in polymer matrices, creating a polymer nanocomposite (PN) [14,16]. In PN systems, the nanoparticles strongly repel each other, hence reducing aggregation possibilities [16]. PNs are exceptionally good for designing novel materials since they are lightweight and easy to process. Optical properties of PNs are also predicted well through recent theoretical calculations, thus providing a solid framework where predictive models work well with the material properties of the polymer and the QDs, considering the chemical properties of the matrices as well as the size effect of the nanoparticles [13]. Optical absorption is a fundamental property of PN systems. Once absorbed, the photons give rise to electronic excitations to higher energy states, causing a subsequent electromagnetic radiation when the excited electrons return to the ground state [1,2,17]. The emitted photons are usually smaller in energies or longer in wavelengths due to Stoke’s shift [1,2,3,14,15,18,19,20]. Although this process is generally consistent, details of how the electronic state changes and the recombination processes could reflect complex events. The changes could occur due to many factors, including but not limited to, scattering and defect or impurity states, interaction between the fluorescing components, environments where the QDs are dissolved/embedded, and interfaces to which they are exposed [1,2,3,6,7,8,17,18]. The mechanism of detailed balance also plays a role in some cases, as has been noted previously [2]. Thus, it is important to characterize photoluminescence from PNs, especially when they are in different chemical environments and/or subjected to varying interfacial interactions.

One recent avenue of exploration involves leveraging QDs as multiplex sensor probes, enabling the generation of photons at multiple wavelengths from a single excitation wavelength [14,16]. The photoluminescent emission from QD systems exhibit lifetimes ranging from nanoseconds to picoseconds, making them suitable for rapid temporal responses. Employing nanoparticles of varying sizes for doping different components of heterogeneous materials allows for the creation of a matrix with low scattering and high photoluminescence (PL) signals. Composite materials, tagged with distinct QDs, exemplify economical, flexible, yet efficient systems. Light-emitting diodes (LEDs), photovoltaic devices, photocatalysis, gas sensing, nonlinear optical devices, non-volatile memories, and tissue engineering, among other applications, has been achieved through II-VI PN materials [1,13,14,15,16]. Among them, CdSe PNs have enjoyed significant attention for their suitability in simple synthesis recipes and versatility. Due the expansive work being performed on device applications in CdSe PNs, it is important to understand the absorption/emission properties of these materials when they are packed together with different host matrices, and/or they are deposited on a solid surface.

Until recently, the influence of QD systems on host materials had not been thoroughly investigated. Earlier reports indicated that the presence of PbS QDs, hetero paired with an undoped GaAs substrate, altered the PL properties of the latter [2,19]. With the rapidly advancing synthesis of composite materials, it becomes crucial to study the interactions and mechanisms altering the optical properties of one or both hetero-paired components.

In this study, we present the synthesis of CdSe QD-doped polymer materials with two different PL emission wavelengths. The PL intensities of the two peaks, originating from differently sized QDs in the system, were tunable by adjusting the ratio of doped silica to polyvinyl alcohol (PVA) microparticles. Laser excitation intensity was varied, and the PL from the two-colored QD system was measured. Analysis of the data revealed two distinct trends in PL intensity growth as a function of laser excitation. Importantly, the trends observed in the composite material differed from those studied previously with hetero-paired QDs deposited on semiconductor substrates, as discussed herein. This early report on novel two-phase orange- and red-emitting PVA-based nanocomposites highlights the appeal of the system for its potential use in sensing and imaging applications and its photoinduced optical limiting properties at the nanoscale.

## 2. Materials and Methods

**Figure 1:**
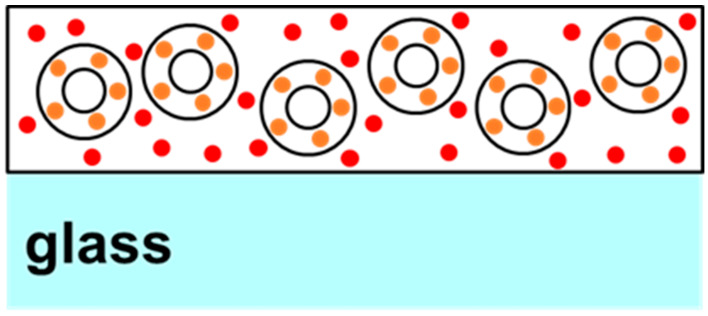
Structure of two-phase two-QD samples showing orange QDs bound in the shells of silica microparticles and red QDs dispersed in the surrounding PVA polymer.

Two-phase quantum dot (QD) films were created by initially suspending the desired mass of “orange” QD-doped silica microparticles emitting at approximately 578 nm in 2.3 g of the 5% polyvinyl alcohol (PVA) solution. For 50% silica film, 0.1 g of microparticles were added, and for 25% silica film, 0.04 g of microparticles, and so forth. A more detailed description has already been reported previously; hence, a short description will be included here [16]. To produce silica microparticles doped with quantum dots, 0.1 g of commercially available silica microparticles (Sigma-Aldrich, St. Louis, MO, USA) was suspended in 3 mL of stock ammonia solution mixed with 4 mL of ethanol and 2 mL of distilled water in a 20 mL vial. To this solution, 10 mg of quantum dots (QDs) emitting light at 578 nm and treated with the surface ligand (3-Mercaptopropyl) trimethoxysilane (provided by Mesolight, Inc., Suzhou, China) are added, along with 0.4 g of tetraethyl orthosilicate (TEOS). The vial was sealed, and the mixture was stirred at room temperature for 1 h. After allowing the reaction mixture to settle, the supernatant was examined for coloration. If coloration persists, an additional 0.1 g of TEOS is introduced, and the reaction mixture is stirred for another hour. This cycle was repeated until the supernatant became visibly clear. At this point, 0.1 g of aminopropyltriethoxysilane (APTES) and 0.1 g of distilled water were incorporated into the reaction vessel, and the solution was stirred for 2 h. The particles were subsequently gathered through centrifugation, washed once with isopropanol, and transferred to a 10 mL vial for drying under vacuum. After adding the microparticles to the polymer solution, the combination was thoroughly mixed to ensure proper dispersal. Subsequently, “red” water-soluble quantum dots emitting at ~650 nm were added to the solution in 10 μL aliquots and mixed. After each addition of red QDs, a drop of the solution was placed onto a microscope slide and dried under vacuum for testing the PL ratio from the two peaks at 50 mW of laser power. In this text, the “orange” and “red” emissions will be sometimes referred to as “peak 1” and “peak 2”, respectively. Figure 1 presents a not-to-scale schematic diagram or cartoon of the cross section of a typical sample thus produced. As can be seen in Figure 1, the “orange” QDs are linked to silica, whereas the “red” QDs are dispersed in the polymer matrix. Once the desired peak intensity ratios were obtained, the particle suspension produced by the above method was drop-cast onto a 2 × 2-inch slide and allowed to cure to form a solid film. A total of four samples were thus created for a systematic study.

The relative emission intensities of the two quantum dots present were then examined by illuminating the dried spot of polymer using a 405 nm collimated continuous-wave (CW) diode laser for excitation. Table 1 presents the four samples studied in this work, where PVA1 corresponds to “orange”:“red” intensity ratio of 4:1, PVA2 corresponds to 2:1, and so forth. The emitted light was observed using a home-built laser fluorescence setup in the backscattering geometry, consisting of a fiber-coupled spectrometer (Silver Nova from StellerNet Inc., Tampa, FL, USA). The excitation laser intensities were adjustable through a calibrated average power vs. diode current curve, ranging from 0 to 315 mW with 3% or less intensity fluctuations, monitored by a computer-controlled power meter form Laserglow Technologies (North York, ON, Canada). The laser powers were then converted to intensities as follows:

intensity (W/cm^2^) = output power (W)/area of laser beam (cm^2^), (1)

where “output power” is defined as the power measured at the sample plane, and “Area” refers to the area covered by the laser beam at the same plane. Unless otherwise stated, exposure time was 5 s and area of the beam was estimated as 0.1257 cm^2^ for all measurements reported here. All experiments were conducted at room temperature. Thus, PL measurements were conducted as a function of increasing 405 nm laser intensity between 0–2.5 W/cm^2^ range.

**Figure 2:**
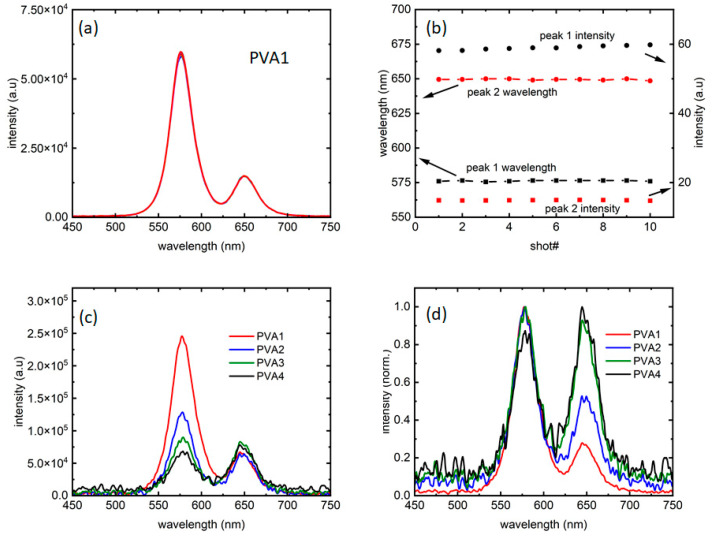
(**a**) A total of 10 traces collected over a period of 1 min show the repeatability of the data with each color presenting one measurement (also referred to as a “shot”). (**b**) Peak 1 and 2 emission wavelengths, along with the corresponding intensities from (**a**) with the arrows showing the axes used. (**c**) Intensity comparison of the four samples studied and (**d**) their normalized versions compared to probe any difference in linewidths.

Figure 2a presents a collection of 10 measurements, taken at an interval of 10 s, confirming that the fluorescence did not change for at least a minute; thus, the intensity of emission was stable for the entirety of the period needed to collect data. Figure 2b confirms that the intensity and peak wavelength fluctuations for both the peaks are minimal, and so are not contributing to the analysis. Figure 2c,d present fluorescence from all the four samples at 0.23 W/cm^2^ intensity, along with its normalized version, respectively.

The process of adding red QD aliquots, drying test samples, and checking emission intensity was repeated until the relative intensities of the emitted peaks of the two quantum dots reached the desired ratios. Once achieved, the particle suspension was drop-cast onto a 2 × 2-inch glass slide and allowed to cure.

In this study, the emphasis is on the QD-doped silica: PVA films where the intensity ratio was varied. A total of 4 samples were prepared and investigated for the purpose of this study. All samples started emitting at a laser power of 0.014 W/cm^2^. Details of the samples are listed in the Table 1.

Hence, as could be seen in Figure 2c, the four samples explored the ratios of intensities in such a way that the “red” dots are more than half of (PVA4), equal to (PVA3), half of (PVA2), and one-fourth of (PVA1) the PL intensities in the samples. As Figure 2d shows, there are almost negligible changes in PL linewidth between the four samples. However, with the introduction of the “red” dots, the PL showed more noise for excitation intensities close to PL threshold. While the reason is unknown at this moment, it could be further evidence of the “red” dots influencing the “orange” dot PL.

## 3. Results and Discussion

**Figure 3:**
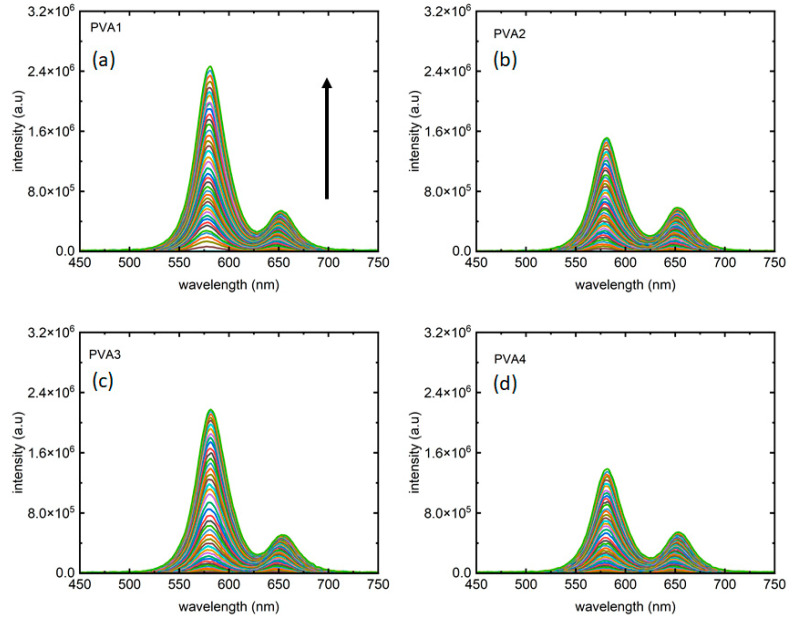
Fluorescence from the two-phase materials as the excitation laser intensity varied from 0–2.5 W/cm^2^. (**a**) PVA1, (**b**) PVA2, (**c**) PVA3, and (**d**) PVA4, respectively, present PL from the four samples, with each color corresponds to an excitation intensity for which the PL has been collected. The arrow shows the direction of increment of excitation laser intensity in all plots.

Figure 3 depicts the two-colored photoluminescence (PL) measurements. As anticipated, distinct emission peaks emerge at “orange” and “red” wavelengths. Each color from the measurements reported in Figure 3a–d corresponds PL collected for an excitation intensity of 405 nm laser. In line with expectations in excitation-dependent measurements, PL intensity increases with rising laser intensity in both peaks. As presented in Table 1 the four samples showed different peak intensity ratios, presented in Figure 3a–d, for PVA1, PVA2, PVA3, and PVA4, respectively. It is noteworthy that not only the peak 1:peak 2 intensity ratios but also the maximum PL intensities for peak 1 from all the four samples are different, with PVA1 and PVA4 marking the largest and smallest maximum intensities for peak 1, respectively.

**Figure 4:**
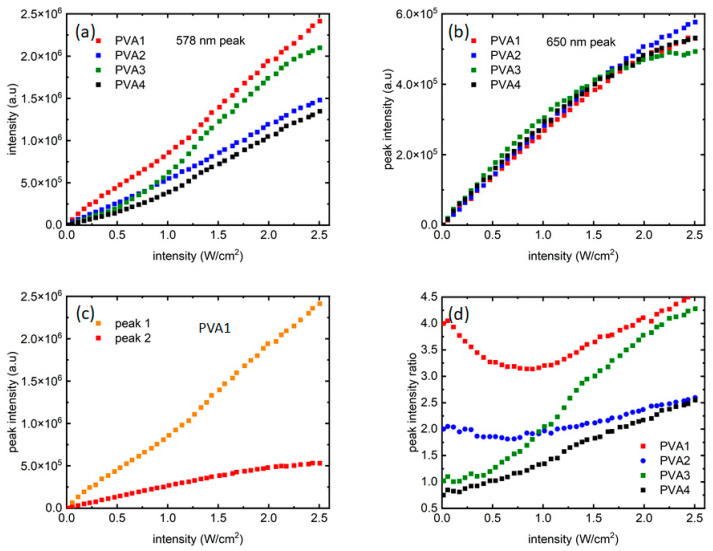
Growth of fluorescence intensities from the two QDs as the excitation laser intensity varied from 0–2.5 W/cm^2^: (**a**,**b**) present intensity growth of peak 1 and peak 2, respectively; (**c**) presents a comparison between peak 1 and peak 2 growth for PVA1. (**d**) Ratio of the two peak intensities as the laser intensity increased.

While the four samples started with certain PL peak intensity ratios, they evolved differently with the increasing laser intensities. To investigate that, peak intensities from the two distinct emission peaks were plotted for all samples as a function of laser intensities. Figure 4a shows the growth of peak 1 (~578 nm, “orange” peak) for all the samples. In Figure 4a, for all the four samples, the peak 1 intensity increased almost linearly, and is very reflective of the increment of laser intensities. PVA3 shows a somewhat different, slightly nonlinear trend. In PVA3, peak 1 showed a nonlinear increase in PL intensities beyond 1 W/cm^2^ and departed from PVA2 around that point, as seen in Figure 4a. The dispersion of QDs in the polymer solution and controlling their relative intensities through dilution is a challenging process, and this might have contributed to a local distortion as well. The only other difference between the two samples is the number of red dots, where an equal number of red and orange dots are present. This could also lead to this anomalous effect. A systematic study using multiple polymers and multiple QDs would be ideal to clarify further, which is beyond the scope of this work but is being undertaken as a future work. In Figure 4b, the growth for peak 2 (~650 nm, “red” peak) can be seen, where it is like peak 1 only up to ~2 W/cm^2^ of laser intensity, after which the peak intensity saturates for all the samples, with a hint of resumption of growth for the last few data points. Figure 4c shows this difference in PL intensity growth mechanisms more closely through a comparison of peak 1 and peak 2 growth data vs. laser intensity for PVA1. Compared to peak 2, peak 1 grew almost an order of magnitude more during the same excitation intensity range. Also, there seems to be a transition from a shallower to a steeper slope in peak 1 intensity growth (Figure 4a) present for all samples. The transition seems to be around ~1 W/cm^2^. It noteworthy that around the same intensity value, peak 2 intensity growth slows down before finally saturating at ~2 W/cm^2^.

Figure 4d shows the way the peak intensity ratios varied when laser intensity increased. It is noteworthy that the growth dynamics of the two QD peaks are sensitive to the starting ratio of the samples. The ratio did not remain the same at all levels of excitation intensities and could be noted as evidence of influence of the “red” dots on the PL growth of the “orange” dots. The difference in peak intensity ratios as the laser excitation increased is a reflection of the way the two peaks grew with excitation intensities, and could be attributed to the continuous growth of peak 1 when peak 2’s eventual saturation.

**Figure 5:**
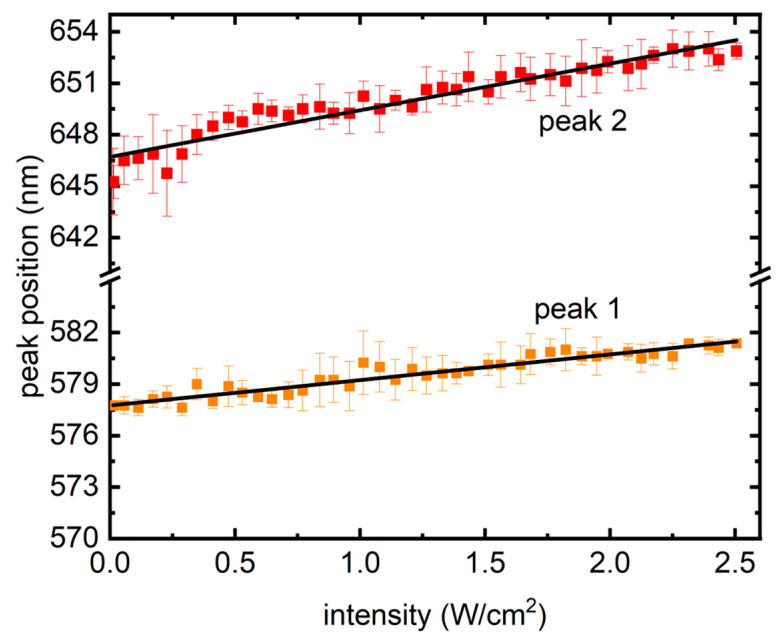
Redshift from the two peaks as the intensity of excitation grew. The symbols represent the average of peak wavelength positions from the four samples, whereas the error bars present their standard deviations. Solid lines are linear fits.

Finally, in Figure 5, peak wavelengths from the traces are shown with increasing laser intensity, where a linear redshift could be seen, which is different from previous reports in semiconductor QDs, where a blue shift was presented owing to the photodynamic Burstein–Moss shift (BMS), also known as dynamic band filling [21]. BMS is a chemically inert doping process and takes place in semiconductors at high degrees of electron–hole (e–h) pair densities. Creation of e–h pairs, in such scenarios, fills the band bottoms, causing a band gap increase and resulting in a nonlinear blue shift in the PL [21]. In contrast, Figure 5 presents a linear red shift in the PL, signifying a shrink in the band gap.

The difference in growth of PL in the two QDs and the way they varied with the starting ratio suggests a strong dependence on the amount of “red” QDs. The nanocomposite samples have certain closely packed QDs held together by a PVA matrix. It is possible that there are interactions and energy transfer processes occurring. One of the mechanisms through which differently sized, closely packed QDs interact is Forster resonance energy transfer (FRET). FRET occurs when there is an overlap between donor emission and acceptor absorption spectra and is a result of dipole–dipole interaction [22].

FRET usually occurs in samples that are homogenous and closely packed with a certain threshold of inter-dot distance. In this work, the two sized dots are not dispersed similarly in the polymer matrix. The dispersion geometry of the two dots is critical for FRET efficiency. As shown in Figure 1, the orange dots are bound in silica shells in these samples, while the red dots are dispersed in the polymer matrix. It has also been reported that FRET is extremely sensitive to temperature and is usually detected at cryogenic temperatures [22]. While it is possible that FRET is present in the measurements reported here, it cannot be the sole mechanism responsible for the properties of the PVA-based PN samples studied here. As presented in Figure 4a,b, the smaller dots (orange peak) are linearly increasing and there is no sign of quenching in them, whereas the larger dots (red peak) saturate and quench around 2 W/cm^2^ intensity of excitation, irrespective of the number of red dots present in the systems. If present, it is more likely that FRET is the dominant mechanism in intensities up to ~1 W/cm^2^. As seen in Figure 4a,b, until 1 W/cm^2^, the orange peaks grow with a shallow slope, while the red peaks show a sharp, linear growth. This also explains the trends of the two peak ratios in Figure 4d, which initially stay flat or decrease before almost linearly increasing. Confirmatory measurements using systematic lifetime measurements in mono-dispersed vs. multi-dispersed QDs samples, along with adequate microscopic information highlighting the inter-dot separation, would be interesting and could be addressed in a future work.

These results are different from a previously reported study on QD absorption or PL detailed balance, where one type of QD solution was drop-cast on different substrates [2]. In that study, all samples showed saturation in PL, well before the maximum laser power presented in the current work [2]. However, the underlying mechanism could still have some similarities between the study in [2] and the current work. It is possible that beyond intensities ~1 W/cm^2^, the process is dominated by the presence of the red dots. The high-energy (405 nm) laser is pumping the orange dots (578 nm) well above the band gap. CdSe QDs fluorescing at 625 nm and above could have reasonable absorption at 578 nm wavelength due to the exciton transition at ~580 nm, which makes analyzing the 650 nm peak more complex and raises the possibility of simultaneous excitation from the 405 nm laser and 578 nm emission from the orange dots triggering more PL from the 650 nm peak [23]. Also, while relaxing down to ground state, the excess energy from the orange dots could be absorbed by the red dots (650 nm). This extra energy can excite the red dots more and thus can cause their saturation. Also, the red dots do not have any active layer beneath them working as potential absorbers; hence, they saturate like the usual QD films studied in [2,18,19,20].

It has been previously reported that both the PL intensity and the spectral shift of PL emissions in polymer-embedded QD systems could be affected by the properties of the polymer matrix [16]. In such materials, the intensity loss/growth were attributed to the e/h transfer processes, whereas the spectral shift was attributed to the temperature and pressure of the samples [16]. It is possible that the soft PVA matrix has a role in the evolution of PL emissions recorded here.

## 4. Conclusions

In this study, we present findings on the optical emission from a two-color quantum dot (QD)-doped silica and polymer system through photoluminescence (PL) measurements. The thin films based on QDs could be excited using a single wavelength in the visible range, emitting at two distinct peaks with controllable intensities depending on the ratio of QDs doped into the silica and polymer. Analysis of the increase in the emission intensities of the two peaks as a function of excitation intensity was conducted, and ideas regarding the mechanism and applications were explored.

The two peaks in the two-phase system exhibited different growth patterns with increasing laser excitation intensities. The peak corresponding to the shorter wavelength (peak 1) displayed nearly linear growth as the excitation intensity increased. However, the linear growth showed a transition point around ~1 W/cm^2^ excitation intensity, where the slopes became steeper, as showcased in Figure 4a.

In contrast, the longer wavelength peak at ~650 nm (peak 2) demonstrated linear growth until a laser intensity of ~1 W/cm^2^, followed by slower, nonlinear growth, before finally saturating around ~2 W/cm^2^. Importantly, these characteristics differed from those observed in PbS or perovskite QD samples on various solid substrates, either crystalline or amorphous [2,18]. The property of changing rates serves as an intriguing characterization tool for QD-based materials and could potentially be linked to the presence of the polymer matrix, the number of phases, and the laser excitations used. Experiments are underway to delve further into the unique features of quantum dots and to study the possibilities of interacting QDs when more than one component is present. One possible explanation for the difference in PL saturation could be a combination of FRET with the high-energy pumping and subsequent relaxation from orange dots occurring at two different excitation intensity regimes. The 405 nm pump could excite the orange dots to a much higher energy than the band before they start relaxing back while giving off the excess energy, leading to absorption of that excess energy by the red dot layer. Last but not least, the presence of PVA plays a role especially in determining the spectra shift.

Some of the differences in PL growths in the four samples were not expected (for example, peak 1 growth in PVA2 vs. PVA3) and were challenging to explain due to the early nature of this report and the novelty of the samples studied. However, possible causes could be suggested which include local distortion in samples, the number of dots present, or a photophysical process not yet considered in the scope of the present study. The results showcase potential for sensing applications. Essentially, this is an early report of a novel multi-phase nanocomposite system eligible for easy integration in polymers, showing some optical limitation stemming from linear and nonlinear PL growths under certain excitation intensities. Researchers have explored ideas in mesoscale sensing, pressure sensitive nanoparticles, and photovoltaic applications in polymer-embedded QD systems, which makes these materials appealing and worthy of further attention [16,24].

## Figures and Tables

**Table 1:** Details of the samples investigated in this study.

Samples	Peak 1 and 2 Intensity Ratios at 405 nm, 0.014 W/cm^2^ Intensity
PVA1	4:1
PVA2	2:1
PVA3	1:1
PVA4	3:4

## Data Availability

The data presented in this study are available within the article.
